# Multi-Target Protective Effects of *Sanghuangporus sanghuang* Against 5-Fluorouracil-Induced Intestinal Injury Through Suppression of Inflammation, Oxidative Stress, Epitheli-Al-Mesenchymal Transition, and Tight Junction

**DOI:** 10.3390/ijms26073444

**Published:** 2025-04-07

**Authors:** Jaung-Geng Lin, Yu-Wen Sun, Wen-Liang Wu, Wen-Ping Jiang, Fang-Yu Zhung, Guan-Jhong Huang

**Affiliations:** 1 School of Chinese Medicine, College of Chinese Medicine, China Medical University, Taichung 404, Taiwan; jglin@mail.cmu.edu.tw (J.-G.L.); boss-16888@hotmail.com (W.-L.W.); 2 Chinese Medicine Research Center, China Medical University, Taichung 404, Taiwan; 3 Department of Chinese Pharmaceutical Sciences and Chinese Medicine Resources, College of Chinese Medicine, China Medical University, Taichung 404, Taiwan; 4 Department of Pharmacy, China Medical University, Taichung 404, Taiwan; wpjiang@cmu.edu.tw; 5 Department of Nutrition, China Medical University, Taichung 404, Taiwan; 6 Department of Food Nutrition and Healthy Biotechnology, Asia University, Taichung 413, Taiwan

**Keywords:** *Sanghuangporus sanghuang*, 5-fluorouracil, mucositis, oxidative stress, inflammation, apoptosis, epithelial–mesenchymal transition, tight junction

## Abstract

Sanghuang (*Sanghuangporus sanghuang*, SS) is a medicinal fungus with multiple pharmacological effects, including antioxidant, anti-inflammatory, immune-boosting, and anti-cancer activities. 5-fluorouracil (5-FU) is a commonly used chemotherapeutic agent for the treatment of colorectal cancer. It primarily exerts its antitumor effect by inhibiting DNA and RNA synthesis, leading to cell apoptosis. However, it frequently induces adverse effects These issues limit the clinical application of 5-FU. This research aims to determine the potential of SS as a therapeutic agent in reducing 5-FU-induced intestinal mucositis in a mouse model. The results indicated that 5-FU administration significantly increased diarrhea severity, reduced colon length, caused small intestinal villus atrophy, disrupted intestinal architecture, led to insufficient crypt cell proliferation, and resulted in weight loss. It also significantly upregulated inflammatory responses, apoptosis, oxidative stress, and epithelial–mesenchymal transition (EMT) pathways, and disrupted the integrity of intestinal mucosal tight junction, while elevating pro-inflammatory cytokines and reducing antioxidant capacity. However, SS significantly ameliorating alleviating the adverse impacts of the chemotherapeutic agent on the intestinal mucosa. In conclusion, this investigation provides the first evidence of the protective effects of SS on 5-FU-induced mucositis. These findings suggest SS as a potential therapeutic application, offering a promising strategy for reducing the adverse effects of 5-FU chemotherapy and improving the treatment and quality of life for colorectal cancer patients.

## 1. Introduction

Chemotherapy, radiation therapy, and surgery are the primary cancer treatments, with chemotherapy emerging as a novel method offering improved survival rates. One of the primary limitations of chemotherapy for cancer is the nonspecific targeting of drugs, which can harm normal tissues. The administration of high-dose anti-cancer drugs during chemotherapy compromises the body’s biomolecular defenses, impairing immunity [1]. The adverse effects of chemotherapy drugs extend to normal cells, especially rapidly dividing oral, gastric, and intestinal mucosal cells, with the intestinal lining suffering the most damage. As a result, the development of intestinal mucositis may cause injury to the intestinal lining and provoke inflammation [2]. Patient susceptibility to intestinal mucositis varies with chemotherapy drug selection and dosing. Approximately 50–80% of patients treated with specific drugs experience symptoms such as gastrointestinal bleeding, diarrhea, abdominal discomfort, malnutrition, and increased infection risk [3]. Intestinal mucositis predominantly targets intestinal epithelial cells with high proliferative capacity, inducing sequential events like cell infiltration, programmed cell death, repair, and regeneration, all of which contribute to inflammation and epithelial barrier damage [4]. Patients suffering from intestinal mucositis experience a notable decline in life quality and treatment efficacy. Crucially, the condition is a significant contributor to lower survival rates and premature mortality associated with certain cancer therapies.

5-Fluorouracil (5-FU) is a commonly administered chemotherapy agent for treating cancers such as colorectal, gastric, breast, and head and neck malignancies. It belongs to the class of antimetabolites and works by interfering with DNA and RNA synthesis, targeting rapidly dividing cancer cells [5]. However, 5-FU can also harm normal, fast-proliferating cells, such as those in the gastrointestinal tract, causing side effects like intestinal mucositis, diarrhea, and immune suppression. Its combination with other drugs or supportive therapies often aims to enhance efficacy and reduce toxicity [6]. To improve the therapeutic potential of 5-FU and minimize associated side effects, novel compounds are needed to treat intestinal mucositis. Current treatments include supportive care, aimed at symptom relief through protective agents, pain medications, and antibiotics [7]. Therapeutic strategies for intestinal mucositis include mucosal protectants, immunomodulatory drugs, growth-promoting factors, and anti-inflammatory medications. Complementary approaches, such as Chinese herbal remedies, dietary supplements, and enzymes, have also shown promise in managing this condition [8]. While no individual approach ensures total efficacy, several agents, including Palifermin (a growth factor), Amifostine (a free radical scavenger), probiotics like *Lactobacillus* spp., zinc sulfate, and antioxidants such as cysteine and L-glutamine, have been proven effective in specific scenarios [9].

The formation of mucositis, a typical side effect of chemotherapy, unfolds through stages including initiation, primary injury response, amplification, ulceration, and healing. DNA strand breaks and ROS generation during initiation specifically harm the basal epithelial and submucosal layers [10]. In the primary damage response, damage to DNA and non-DNA molecules initiates ROS activation pathways like NF-κB. This leads to signal amplification through transcription, causing tissue damage and an accumulation of inflammatory cytokines. During ulceration, mucosal structures collapse, resulting in painful injuries that allow microorganisms to enter and cause infections [11]. Healing occurs as epithelial cells migrate, proliferate, and differentiate to restore the ulcer site. Cytotoxicity from drugs halts DNA synthesis and thymidylate production, while ROS production initiates apoptosis. The simultaneous activation of NF-κB stimulates excessive pro-inflammatory cytokines, leading to aggravated tissue damage [9].

A medicinal polypore bacterium, *Sanghuangporus sanghuang* (SS), is commonly used in Taiwan, Japan, China, and Korea. Its pharmacological benefits include neuroprotection, liver protection, antioxidative stress reduction, anti-cancer activity, anti-inflammation, antiviral effects, anti-diabetic properties, and immune modulation [12,13,14,15]. Effective prevention and treatment options for intestinal mucositis remain scarce. This research focuses on the impact of SS on 5-FU-induced intestinal mucositis and delves into the mechanisms involved. Understanding the impact of SS on 5-FU-induced colitis may offer stronger theoretical support for its potential benefits to intestinal health.

## 2. Results

### 2.1. The Role of SS in Modulating Physical Symptoms, Including Weight Loss, Diarrhea, and Intestinal Length, Was Assessed in the 5-FU-Induced Intestinal Mucositis Model

**Figure 1:**
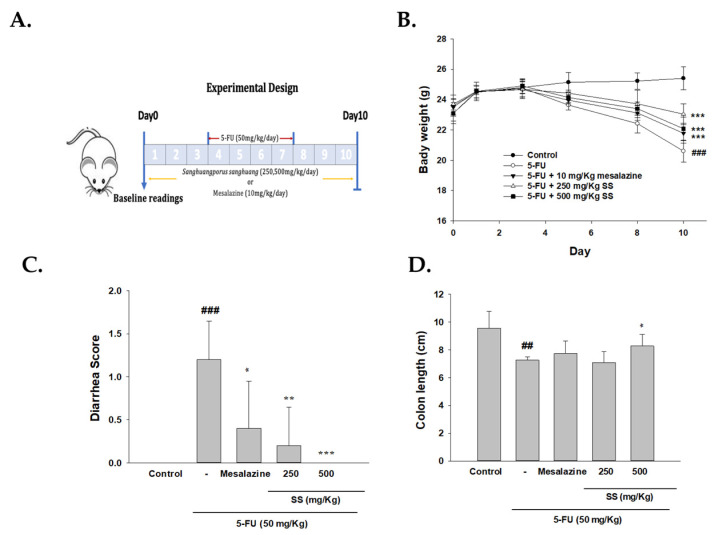
The administration of SS resulted in a reduction of 5-FU-induced intestinal mucositis in mice. The experimental design (**A**) included measurements of body weight (**B**), diarrhea (**C**), and the length of the entire intestine (**D**) for each group. SS at 250 and 500 mg/kg was given daily for 10 days, while 5-FU was administered between days 4 and 7. Mice were sacrificed on the 10th day. Data are expressed as mean ± S.D. (*n* = 5). ### *p* < 0.001 compared to control group; ## *p* < 0.01 compared to control group. * *p* < 0.05, ** *p* < 0.01 and *** *p* < 0.001 compared to 5-FU group.

Weight loss and diarrhea were the main metrics for evaluating the health status of the mice. Continuous monitoring for mucositis-related physical signs was performed during the entire experimental phase. Mice in the control group maintained smooth fur, normal dietary intake, hydration, and active movement throughout the study, with no evidence of diarrhea. In contrast, those in the 5-FU (50 mg/kg) treatment group began showing adverse symptoms by day 5, including diarrhea, decreased food consumption, reduced activity levels, and unkempt fur. By day 8, their condition deteriorated further, presenting with severe, blood-tinged watery diarrhea and a pronounced arched back posture. During days 4 to 7, mice receiving intraperitoneal 5-FU exhibited significant body weight loss, primarily due to severe diarrhea. In contrast, the normal control group demonstrated a gradual increase in weight. Notably, SS administration before the experiment reduced these symptoms in a dose-dependent fashion, with both 250 mg/kg and 500 mg/kg doses yielding substantial improvements (Figure 1B). The marked improvement in 5-FU-induced physical symptoms following SS administration suggests a protective mechanism of SS during the progression of 5-FU-induced intestinal mucositis.

The Bowen scoring system classified diarrhea into four categories based on stool consistency: 0 (normal stool), 1 (slightly wet and soft, indicating mild diarrhea), 2 (wet and shapeless, representing moderate diarrhea), and 3 (watery, indicating severe diarrhea). Scoring began on the first day of 5-FU treatment, with daily assessments noted. On day 10, the SS-treated group was compared to the 5-FU group (Figure 1C). These results underline the substantial protective potential of SS against intestinal mucositis induced by 5-FU.

We first measured the colon length in the various treatment groups to further evaluate the histopathological characteristics. Mice treated with 5-FU had an average colon length of 7.3 cm, significantly shorter than the 9.6 cm observed in the control group. However, the shortening of the colon was alleviated considerably, with an average length of 8.3 cm, in mice treated with SS (500 mg/kg), even under 5-FU treatment conditions (Figure 1D).

### 2.2. Effect of SS on 5-Fu-Induced Intestinal Histopathological Changes

**Figure 2:**
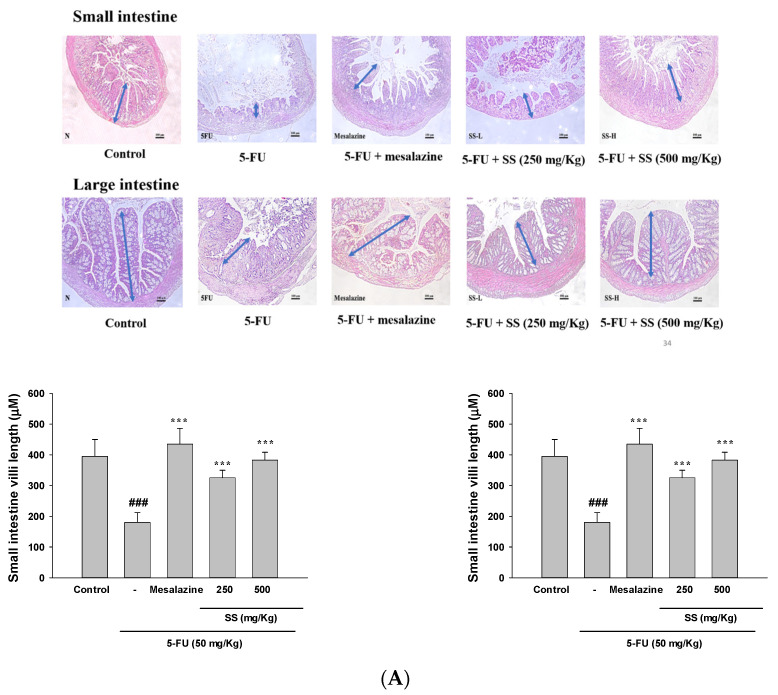

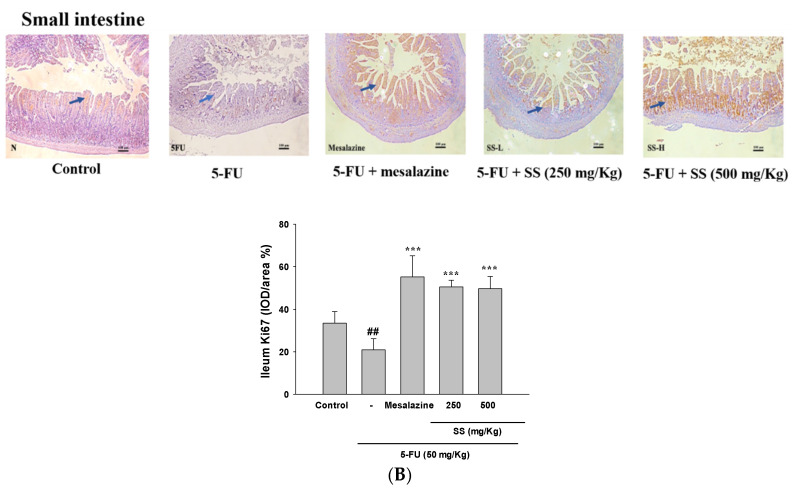
The effect of SS on H&E staining (**A**) and Ki-67 (**B**) expression was analyzed in the intestinal tissues of 5-FU-treated mice. Histopathological observations were made using H&E and Ki-67-stained sections under a light microscope. SS at 250 and 500 mg/kg was given daily for 10 days, while 5-FU was administered between days 4 and 7. Mice were sacrificed on the 10th day. Data are expressed as mean ± SD (*n* = 5). ### *p* < 0.001 compared to control group; ## *p* < 0.01 compared to control group. *** *p* < 0.001 compared to 5-FU group. Magnification, ×100; scale bar, 100 µm.

Mucosal injuries in the small and large intestines were evaluated using hematoxylin and eosin (H&E) staining under a light microscope. In the control group, the villi remained undamaged, showing tall columnar and goblet cells with normal crypt mitosis. However, 5-FU-treated mice exhibited marked histopathological changes, including the total absence of crypt cells, extensive villous atrophy, inflammatory cell infiltration within the lamina propria, and mucosal vacuolization and edema (Figure 2A). Pre-treatment with SS mitigated morphological changes in the small and large intestines, reducing villus blunting, crypt cell apoptosis, and inflammatory cell infiltration. These findings suggest that SS significantly alleviates 5-FU-induced damage in both intestinal regions.

### 2.3. Elevated Ki-67 Levels in the SS-Treated Group Indicated Improved Cellular Proliferation in the Mucositis Model

Ki-67 immunohistochemical staining was used to evaluate the proliferative activity of SS in a mouse model of 5-FU-induced small intestinal mucositis. The control group exhibited strong Ki-67 expression in both the cytoplasm and nuclei of the villi and crypts, while Ki-67 levels were significantly diminished in mice treated with 5-FU alone (*p* < 0.01). Remarkably, the inhibitory effect of 5-FU on intestinal proliferation was effectively reversed by SS treatment, resulting in Ki-67 levels comparable to those of the untreated control group (Figure 2B). The study primarily utilized small intestinal mucosa due to its higher tissue availability compared to other mucosal regions.

### 2.4. The Administration of SS Inhibited the Expression of Pro-Inflammatory Cytokines Triggered by 5-FU

**Figure 3:**
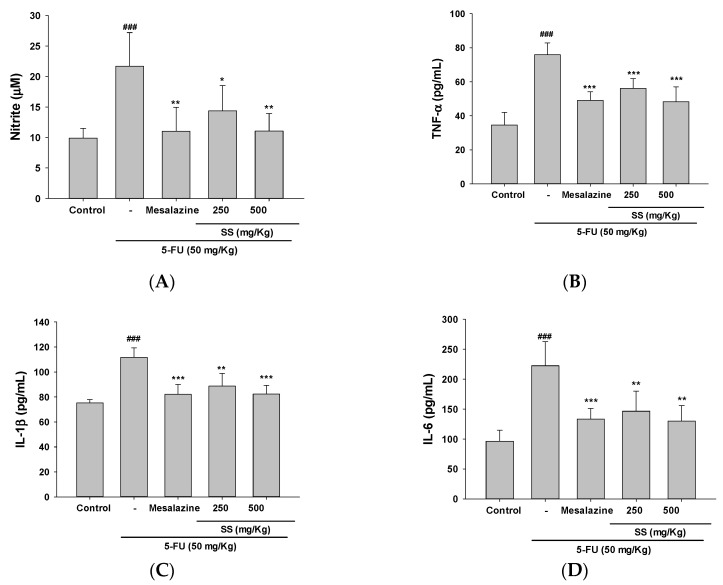
SS mitigated inflammation in a 5-FU-induced intestinal mucositis mouse model, as indicated by serum concentrations of NO (**A**), TNF-α (**B**), IL-1β (**C**), and IL-6 (**D**). Doses of 250 and 500 mg/kg SS were given daily over 10 days, with 5-FU administered on days 4 through 7. Mice were sacrificed on the 10th day. Data are expressed as mean ± SD (*n* = 5). ### *p* < 0.001 compared to control group; * *p* < 0.05, ** *p* < 0.01 and *** *p* < 0.001 compared to 5-FU group.

To understand the mechanism by which SS suppresses 5-FU-induced mucositis, we examined the expression of inflammatory factors in a 5-FU-induced mouse model, with and without SS treatment. Inflammation significantly contributes to mucositis pathogenesis, with pro-inflammatory cytokines identified as key factors and therapeutic opportunities for its treatment. As depicted in Figure 3A–D, NO, TNF-α, IL-1β, and IL-6 concentrations were substantially increased in the 5-FU group relative to controls. Treatment with SS (250 and 500 mg/kg) significantly mitigated these increases, producing cytokine levels comparable to the control group. The inhibitory effect of Mesalazine (10 mg/kg), employed as a positive control, was on par with the response seen in the 5-FU-treated group.

### 2.5. SS Alleviates Oxidative Stress in the 5-FU-Induced Intestinal Mucositis 

**Figure 4:**
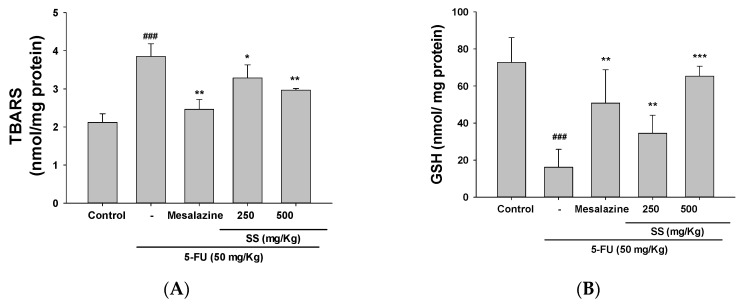
SS demonstrated its capacity to counteract oxidative stress in the intestinal mucosa caused by 5-FU, as determined by MDA (**A**) and GSH (**B**) assays. Data are expressed as mean ± SD (*n* = 5). ### *p* < 0.001 compared to control group; * *p* < 0.05, ** *p* < 0.01 and *** *p* < 0.001 compared to 5-FU group.

Elevated ROS levels are commonly associated with pathological conditions, including oxidative stress and subsequent apoptosis. The study evaluated the impact of SS pretreatment on oxidative stress markers, MDA and GSH, in the small intestines of 5-FU-treated mice. Elevated levels of MDA were observed in the small intestine following 5-FU administration compared to the control group (Figure 4A). SS (250 and 500 mg/kg) pretreatment effectively mitigated the increase in MDA levels caused by 5-FU-induced oxidative stress.

GSH levels, a critical marker of antioxidant activity, were analyzed to assess the protective effects of SS in the small intestines. Treatment with 5-FU led to a marked reduction in GSH levels compared to the control group. Administration of SS at doses of 250 mg/kg and 500 mg/kg significantly improved GSH concentrations in the intestinal mucosa. The ability of SS to restore GSH levels highlights its potential role in managing intestinal mucositis therapeutically. These observations underscore the role of SS in counteracting oxidative damage associated with 5-FU treatment (Figure 4B).

### 2.6. SS Mitigated Inflammation, NF-κB, and the MAPK Signaling Pathway in the 5-FU-Induced Intestinal Mucositis

**Figure 5:**
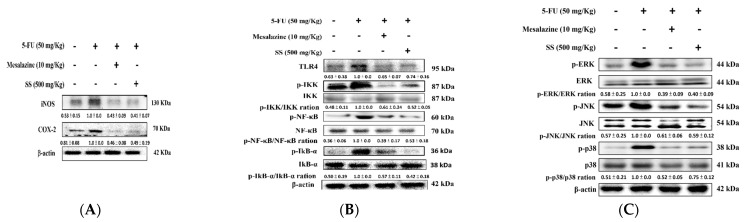
Treatment with SS resulted in the inhibition of iNOS, COX-2 (**A**), TLR4, IKK, NF-κB (**B**), and phosphorylated MAPK (**C**) protein levels in the 5-FU-induced intestinal mucositis model. Western blot analysis measured the protein levels of iNOS, COX-2, TLR4, IKK, p-IKK, NF-κB, p-NF-κB, and phosphorylated MAPK in small intestine tissues. Densitometric analysis was performed to quantify the protein bands. Each experiment was repeated independently at least three times, and representative images were shown. Each result is shown as mean ± S.D. Protein levels were quantified and displayed as fold changes relative to β-actin, which acted as the internal control for normalization.

Inflammation involves the activation of multiple molecular pathways and regulatory factors. SS (500 mg/kg) demonstrated anti-inflammatory effects by significantly reducing COX-2 expression in inflamed tissues, as shown in Figure 5A. The administration of SS at 500 mg/kg doses also decreased iNOS levels in the experimental model. The positive control, mesalazine, effectively reduced iNOS and COX-2 protein levels, supporting its known anti-inflammatory properties. These findings highlight the potential of SS as a therapeutic agent for alleviating inflammation in 5-FU-induced intestinal mucositis.

Toll-like receptors (TLRs) are crucial in recognizing pathogen-associated molecules and triggering immune responses. TLR4 activation was significantly elevated in 5-FU-induced intestinal mucositis, as demonstrated by Western blot analysis (Figure 5B). Pretreatment with SS effectively reduced TLR4 upregulation, suggesting a regulatory effect on inflammatory signaling pathways. The observed suppression of TLR4 signaling by SS underscores its potential as an anti-inflammatory agent. These results support the hypothesis that SS modulates inflammatory responses through the TLR4 axis in 5-FU-induced mucositis.

Essential for the initiation of inflammatory cascades, NF-κB pathway activation is connected to a range of human diseases, particularly inflammatory disorders. Following 5-FU treatment, increased activation of NF-κB, IKK, and IκBα was observed in small intestinal tissues, with Western blot analysis revealing elevated p-NF-κB, p-IKK, and p-IκBα levels. The levels of p-NF-κB, p-IKK, and p-IκBα in 5-FU-induced paw edema were significantly diminished by SS pretreatment, pointing to modulation of the TLR4/NF-κB signaling pathway in acute inflammation (Figure 5B).

In the regulation of inflammatory responses, the mitogen-activated protein kinases (MAPKs) signaling pathway is essential and is implicated in the pathogenesis of many diseases. Exposure to 5-FU resulted in elevated phosphorylation of JNK, ERK, and p38 proteins, which points to the activation of the MAPK pathway within inflamed tissues. Treatments with SS and mesalazine successfully suppressed the phosphorylation of MAPK proteins induced by 5-FU. The observed effects were specific to phosphorylation, as the total protein levels of MAPKs remained unchanged (Figure 5C). These findings demonstrate that SS can effectively inhibit MAPK pathway activation in 5-FU-induced intestinal mucositis, reducing inflammation.

### 2.7. SS Administration in the 5-FU-Induced Mucositis Model Improves Intestinal Antioxidant Defenses and Promotes the Activation of the HO-1/Nrf2 Signaling Pathway

**Figure 6:**
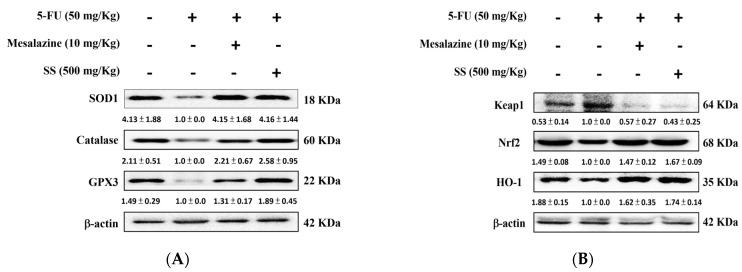
SS modulates the protein expression induced by 5-FU, affecting the levels of antioxidative enzymes (catalase, SOD1, and GPx3) (**A**), as well as Keap1, HO-1, and Nrf2 (**B**) in the small intestine. Western blot analysis was used to quantify the protein expression of these antioxidative enzymes and transcription factors in small intestinal homogenates after 5-FU treatment. Densitometric analysis was performed to quantify the protein bands. Each experiment was repeated independently at least three times, and representative images were shown. Each result is shown as mean ± S.D. Protein levels were quantified and displayed as fold changes relative to β-actin, which acted as the internal control for normalization.

Oxidative stress damages the small intestine and triggers inflammation, resulting in further tissue injury. Figure 6A illustrates that 5-FU reduces antioxidant defenses in the small intestine, as demonstrated by decreased levels of catalase, SOD1, and GPx3. SS pretreatment nearly normalized these antioxidant levels when compared to treatment with 5-FU alone.

Oxidative stress injures intestinal tissue and triggers inflammation, worsening damage by increasing ROS production. ROS overactivity harms lipids, DNA, and other vital macromolecules, leading to cell death. Figure 6B shows that 5-FU increased Keap1 expression while decreasing HO-1 and Nrf2 levels compared to controls. Oral SS administration enhanced Nrf2 and HO-1 expression and decreased Keap-1 compared to 5-FU alone (Figure 6B). These findings suggest that SS can boost antioxidant enzyme expression during 5-FU exposure.

### 2.8. SS Mitigates the Apoptosis Signaling Pathway Induced by 5-FU

**Figure 7:**
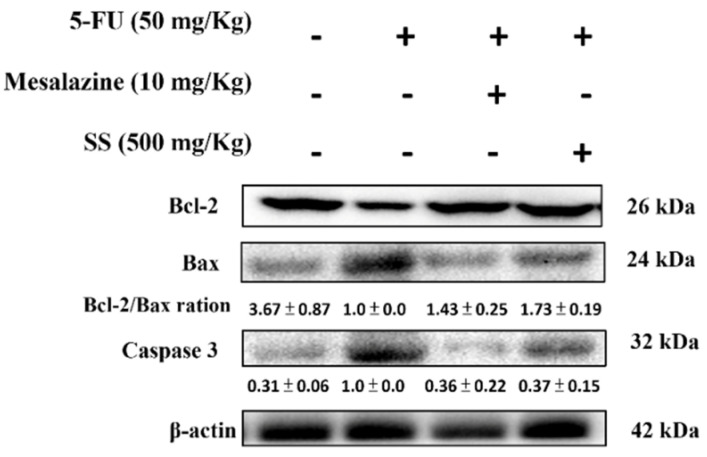
SS administration influenced the expression of Bax, Bcl-2, and caspase-3 proteins in small intestinal tissues after 5-FU exposure. Western blot analysis was conducted using antibodies specific to Bax, Bcl-2, caspase-3, and β-actin. Densitometric analysis was performed to quantify the protein bands. Each experiment was repeated independently at least three times, and representative images were shown. Each result is shown as mean ± S.D. Protein levels were quantified and displayed as fold changes relative to β-actin, which acted as the internal control for normalization.

To investigate the anti-apoptotic effects of SS after 5-FU treatment, immunoblotting was performed to assess the apoptosis-related proteins Bax, Bcl-2, and caspase-3. The 5-FU group displayed higher levels of Bax and caspase-3, alongside lower levels of Bcl-2, compared to the control. However, SS pretreatment reversed these alterations by reducing Bax and caspase-3 levels and elevating Bcl-2 expression (Figure 7).

### 2.9. SS Attenuates the 5-FU-Induced Regulation of the PI3K-AKT Signaling Pathway

**Figure 8:**
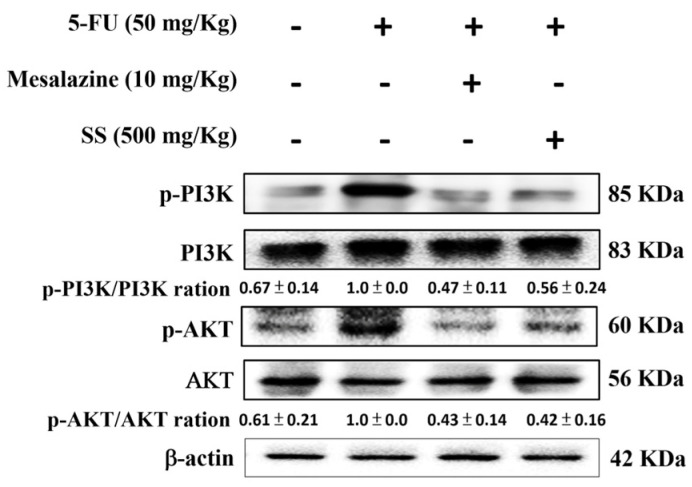
SS attenuated the expression of the PI3K-AKT signaling axis following 5-FU exposure. Homogenized small intestinal samples were subjected to Western blot analysis to measure the protein levels of PI3K, p-PI3K, AKT, and p-AKT. Densitometric analysis was performed to quantify the protein bands. Each experiment was repeated independently at least three times, and representative images were shown. Each result is shown as mean ± S.D. Protein levels were quantified and displayed as fold changes relative to β-actin, which acted as the internal control for normalization.

The PI3K/Akt pathway is critically involved in mucositis pathogenesis as a key regulator of cell growth and oxidative stress in inflammation. According to Figure 8, the 5-FU-only group exhibited a marked increase in PI3K and Akt protein levels compared to the control group. However, SS administration significantly suppressed these levels, demonstrating its role in mitigating mucositis through the PI3K/Akt signaling pathway. 

### 2.10. SS Alleviates the 5FU-Induced Epithelial–Mesenchymal Transition (EMT) Pathways

**Figure 9:**
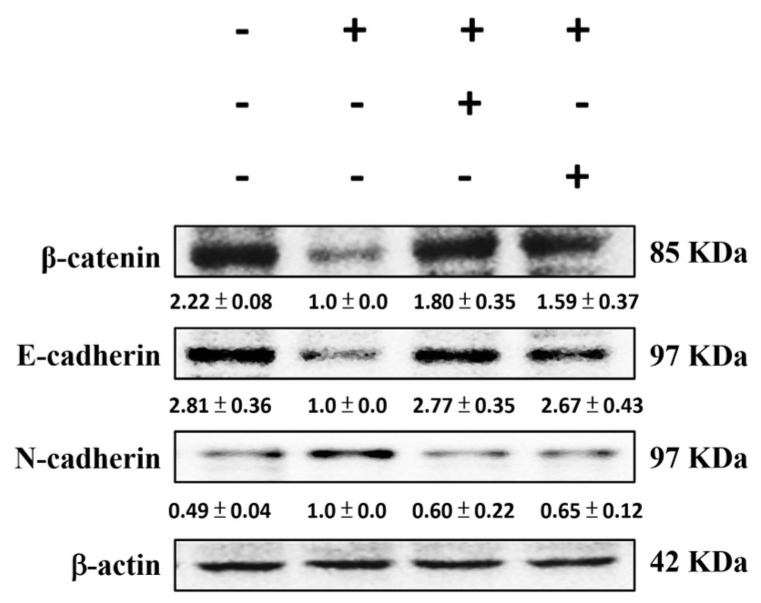
SS influenced the expression of EMT-related proteins in mice with 5-FU-induced mucositis. Small intestinal tissue lysates were analyzed using Western blot with specific antibodies targeting β-catenin, E-cadherin, and N-cadherin. Densitometric analysis was performed to quantify the protein bands. Each experiment was repeated independently at least three times, and representative images were shown. Each result is shown as mean ± S.D. Protein levels were quantified and displayed as fold changes relative to β-actin, which acted as the internal control for normalization.

EMT pathways play a pivotal role in the progression of 5-FU-induced mucositis, a condition marked by severe gastrointestinal epithelial damage. EMT is characterized by a reduction in epithelial markers like cell adhesion and an increase in mesenchymal traits. As depicted in Figure 9, 5-FU exposure reduced β-catenin and E-cadherin protein expression while enhancing N-cadherin levels. However, SS pretreatment restored β-catenin and E-cadherin levels and suppressed N-cadherin expression. These results underscore the potential of SS to inhibit EMT-mediated damage, offering a therapeutic avenue for 5-FU-induced mucositis.

### 2.11. SS Alleviates the 5-FU-Induced Tight Junction Protein Expressions

**Figure 10:**
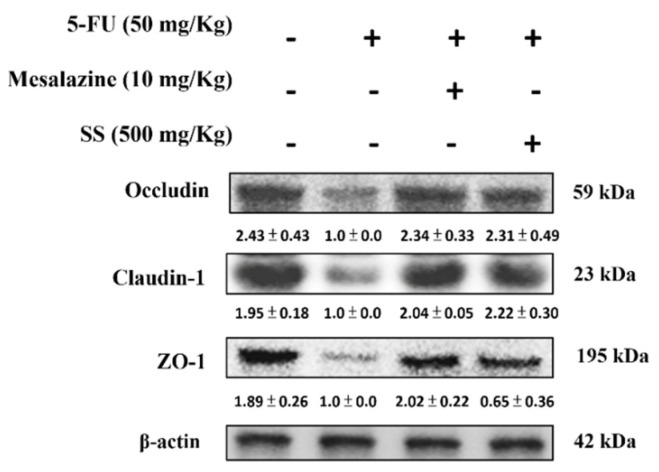
SS influenced tight junction protein expression regulation in mice subjected to 5-FU exposure. Western blot analysis was applied to small intestinal tissue lysates to assess ZO-1, occludin-1, and claudin protein levels using specific antibodies. Densitometric analysis was performed to quantify the protein bands. Each experiment was repeated independently at least three times, and representative images were shown. Each result is shown as mean ± S.D. Protein levels were quantified and displayed as fold changes relative to β-actin, which acted as the internal control for normalization.

5-FU-induced mucositis disrupts the intestinal epithelial barrier by altering the expression of tight junction proteins such as ZO-1, occludin, and claudin-1, essential for cell adhesion and permeability regulation. As shown in Figure 10, exposure to 5-FU significantly reduced the expression of these proteins. Pretreatment with SS was effective in restoring ZO-1, occludin-1, and claudin levels. These results emphasize the role of SS in enhancing tight junction protein expression and its therapeutic potential for treating 5-FU-induced mucositis.

## 3. Discussion

5-FU, an effective chemotherapy drug for colorectal, breast, and head and neck cancers, works by disrupting DNA and RNA synthesis in rapidly dividing cells. Unfortunately, this mechanism also leads to adverse effects such as gastrointestinal toxicity, bone marrow suppression, skin issues, and appetite loss [10]. Gastrointestinal toxicity inflames and ulcerates the intestinal lining, resulting in abdominal discomfort, diarrhea, and malabsorption. Bone marrow suppression lowers blood cell counts, causing anemia and fatigue. Skin-related side effects, including hand-foot syndrome, involve redness and peeling, often with pain. Appetite loss from anorexia and nausea worsens malnutrition during treatment [16,17].

The extent of injury caused by 5-FU in mouse models highly correlates with the administered dose. Higher doses (e.g., 150 mg/kg and above) are associated with severe intestinal damage, systemic toxic effects, and bone marrow suppression, often presenting as significant weight loss, appetite suppression, and diarrhea [18]. In contrast, lower doses (e.g., 50 mg/kg or less) result in milder damage while still inducing conditions like intestinal mucositis. Researchers often vary the dosage of 5-FU in studies to analyze the dose-dependent tissue damage and the efficacy of potential protective therapies. The low-dose model, utilizing less than 50 mg/kg of 5-FU, is designed to simulate chronic or subacute injury with milder clinical symptoms. In contrast, the high-dose model, exceeding 100 mg/kg, serves as an acute injury model to investigate severe intestinal and systemic responses. These dose ranges can be adjusted to meet specific research objectives, enabling the evaluation of therapeutic and protective strategies [19]. Our study showed that mice treated with 50 mg/kg of 5-FU exhibited typical symptoms of intestinal mucositis, including weight loss and diarrhea, consistent with findings from other models, and no mortality was observed. Additionally, our results demonstrated that SS significantly reduced 5-FU-induced diarrhea, supporting observations from the mouse model.

The dosage of 5-FU administered in cancer treatment is determined by several factors, including the patient’s cancer type, age, and renal and hepatic function. Gastrointestinal mucositis is a frequent complication, presenting with symptoms like abdominal pain, malabsorption, diarrhea, ulcers, and bleeding. Diarrhea is particularly detrimental, causing dehydration, malnutrition, and heightened infection risks [5,6]. These issues can result in dose adjustments or even discontinuation of chemotherapy, which may compromise its effectiveness and lead to increased treatment duration and healthcare expenses due to prolonged hospitalizations. Chemotherapy-induced intestinal mucositis often leads to decreased food intake, a consequence of reduced intestinal absorptive capacity. This condition is associated with diarrhea and weight loss [20]. Studies report that diarrhea affects 50–80% of chemotherapy patients, frequently becoming a limiting factor in treatment. In this study’s mucositis model, 5-FU administration triggered villous stem cell apoptosis and inhibited proliferation, resulting in shortened intestinal villi. Weight loss was observed on the fifth day of 5-FU administration, likely linked to its toxicity. Conversely, in the 5-FU + SS group, body weight increased from the fifth to the tenth day, suggesting an improvement due to SS’s antidiarrheal effect. While diarrhea appeared in the 5-FU group, it was absent in the 5-FU + SS (500 mg/kg) group, indicating SS’s ability to mitigate diarrhea. These findings suggest that SS alleviates 5-FU-induced mucositis by improving weight loss, diarrhea, and crypt damage. In this study, intestinal mucositis was induced in mice via intraperitoneal injection of 5-FU (50 mg/kg), accompanied by oral administration of SS until day 10. Histopathological analysis revealed that H&E-stained sections from 5-FU-treated mice exhibited shortened villi, crypt damage, and heightened mucosal inflammation. Additionally, Ki-67 staining demonstrated a significant reduction in cellular proliferation, indicative of impaired repair mechanisms commonly associated with 5-FU toxicity [21]. Remarkably, oral SS treatment improved H&E scores, restoring villus and crypt architecture, and enhanced Ki-67 expression, suggesting its protective effects. These findings highlight the potential of SS to mitigate histopathological changes and support intestinal regeneration in 5-FU-induced mucositis.

Cytokines produced by the intestinal immune system are pivotal in preserving intestinal homeostasis, as they modulate immune responses, epithelial barrier integrity, and microbial equilibrium [22]. The delicate balance between pro-inflammatory and anti-inflammatory cytokines governs both protective immune mechanisms and the progression of intestinal pathologies, including mucositis [23]. In our study, 5-FU administration disrupted this cytokine equilibrium. However, SS treatment markedly suppressed TNF-α, IL-1β, and IL-6 expression levels, indicating its efficacy in mitigating intestinal mucosal damage by re-establishing cytokine homeostasis. Cancer treatment often leads to mucosal injury, primarily driven by the release of pro-inflammatory cytokines and oxidative stress. These mechanisms play a central role in the development of intestinal mucositis, a common and debilitating complication of radiation and chemotherapy [24]. The chemotherapy drug 5-FU generates ROS as part of its mechanism of action. ROS can directly damage cellular components such as DNA, lipids, and proteins, resulting in epithelial cell death and disrupting the intestinal barrier function. The damage to epithelial cells triggers the production of inflammatory mediators, worsening tissue injury, and contributing to mucositis development. TNF-α, IL-1β, and IL-6, vital mediators of inflammation, are released by immune cells, endothelial cells, and epithelial cells following oxidative stress and cellular injury [25]. The inflammatory cascade triggered by these cytokines includes increased vascular permeability, immune cell recruitment to the injury site, and enhanced tissue damage. By disrupting tight junction proteins and promoting epithelial cell apoptosis, these cytokines impair the intestinal barrier, resulting in malabsorption, diarrhea, and a higher susceptibility to infections. The relationship between oxidative stress and the release of pro-inflammatory cytokines is bidirectional [26]. Oxidative stress can trigger the release of cytokines, which, in turn, exacerbate oxidative damage. For instance, TNF-α has been shown to elevate ROS production, creating a vicious cycle of intestinal inflammation and cellular damage. This interaction plays a key role in the persistent inflammation observed in chemotherapy-induced mucositis and other treatment-related injuries, resulting in severe tissue damage and delayed healing. This causes significant tissue damage and hinders the healing process [27]. Our study confirmed that SS significantly reduced ROS production, lowered pro-inflammatory cytokine levels in the small intestine after 5-FU treatment, and inhibited the associated cytokine signaling pathway. These findings are important for elucidating the mechanism by which SS mitigates 5-FU-induced mucosal damage. 

As a critical player in the innate immune response, TLR4 (Toll-like receptor 4) identifies pathogen-associated molecular patterns (PAMPs) or damage-associated molecular patterns (DAMPs), triggering signaling cascades that lead to inflammation [28]. One of the key markers of TLR4 activation is the phosphorylation of the NF-κB p65 subunit, which plays a significant role in the inflammatory response. Detecting p65 phosphorylation highlights the interplay between TLR4 signaling and inflammatory mechanisms in conditions such as sepsis, cancer, and chronic inflammation [29]. NF-κB-regulated pro-inflammatory cytokines, such as TNF-α, IL-6, and IL-1β, are key drivers of inflammation in 5-FU-induced intestinal mucositis. Elevated cytokine levels in the ileum of treated mice disrupt epithelial integrity and promote tissue injury. This is mediated by TLR4/NF-κB activation, which triggers cytokine transcription [30]. These findings highlight potential therapeutic targets to mitigate mucositis severity in clinical settings. Increased NF-κB activation is a hallmark of inflammation, cytokine production, and mucosal damage, as observed in histopathological studies of the small intestine. 5-FU-treated mice displayed significantly elevated levels of TNF-α, IL-6, and IL-1β compared to control groups [31]. SS pretreatment effectively reduced these cytokine levels and NF-κB activation while protecting mucosal integrity. These findings suggest SS achieves its protective effects by inhibiting TLR4 activation and blocking NF-κB p65 phosphorylation.

MAPKs (mitogen-activated protein kinases) are crucial in developing 5-FU-induced mucositis. These kinases, including ERK, JNK, and p38 MAPK, respond to stress stimuli such as oxidative stress, inflammatory cytokines, and chemotherapeutic agents like 5-FU [32]. Their activation regulates critical cellular processes like inflammation, apoptosis, and tissue repair. Research in animal models of 5-FU-induced mucositis shows that increased MAPK activation strongly correlates with heightened inflammation and tissue damage [33]. MAPKs play a critical role in inflammatory signaling pathways that exacerbate 5-FU-induced mucositis. These kinases are closely linked to the activation of inflammatory mediators such as NF-κB, TNF-α, and COX-2, which are key contributors to inflammation and tissue damage. Specifically, p38 and JNK MAPKs facilitate NF-κB activation by phosphorylating upstream regulators like IκB kinase (IKK) [34]. Moreover, MAPKs are directly involved in the transcription and release of TNF-α, a cytokine essential for initiating and sustaining inflammatory cascades in mucositis. ROS generated during 5-FU treatment is further activated. MAPK pathways amplify NF-κB and COX-2 activity and perpetuate a cycle of oxidative damage and inflammation [35]. Our findings demonstrate that SS effectively inhibits MAPK phosphorylation, thereby protecting against mucosal injury caused by 5-FU administration. Additionally, SS prevents the upregulation of pro-inflammatory cytokines by suppressing NF-κB activation in 5-FU-induced models. These results underscore SS’s potential to inhibit the phosphorylation of both NF-κB and MAPKs in response to 5-FU exposure.

5-FU-induced mucositis is strongly associated with oxidative stress, which serves as a major driver of cellular damage and inflammation during chemotherapy. As part of its anti-cancer mechanism, 5-FU generates ROS. While these ROS effectively target cancer cells, they also harm normal cells, particularly those in rapidly dividing tissues like the intestinal epithelium [36]. Elevated ROS levels surpass the capacity of antioxidant defenses, such as glutathione and superoxide dismutase, resulting in oxidative stress. This leads to lipid peroxidation, DNA damage, and protein modification, culminating in epithelial cell apoptosis, intestinal barrier disruption, and mucositis development. The Nrf2-Keap1-HO-1 axis serves as a pivotal defense mechanism against oxidative stress [34]. During 5-FU-induced stress, Nrf2 activation enhances the expression of antioxidant enzymes like HO-1, which protect cells by mitigating ROS-induced damage. The activation of the MAPK pathway by oxidative stress results in increased inflammation and apoptosis within intestinal tissues. Furthermore, oxidative stress aggravates the inflammatory process by stimulating the release of pro-inflammatory cytokines, including TNF-α, IL-6, and IL-1β, thereby amplifying tissue damage and sustaining oxidative stress [37]. The activation of the TLR4/NF-κB pathway by oxidative stress aggravates inflammation, leading to more severe mucosal injuries. Emerging studies indicate that oxidative stress drives histopathological changes by generating lipid peroxidation products and suppressing antioxidant enzyme expression. In response to oxidative stress, Nrf2 is released from the inhibitory Nrf2-Keap1 complex, activating genes involved in antioxidant pathways. This process upregulates antioxidant proteins, such as HO-1, GPx, and GSH-S-transferase, which are crucial for protecting tissues from the harmful effects of free radicals [38]. Experimental evidence from our study highlights that SS modulates the Nrf2-HO-1 axis, protecting against oxidative stress induced by 5-FU.

Apoptosis is a fundamental process in the development of 5-FU-induced intestinal mucositis. As an anti-cancer agent, 5-FU disrupts DNA and RNA synthesis in rapidly dividing cells, affecting not only tumors but also normal epithelial cells. In the intestines, this disruption causes the loss of crypt cells, which are essential for tissue repair and regeneration [39]. Within the intestinal epithelium, 5-FU activates apoptotic pathways, leading to the destruction of crypt stem cells. This results in impaired cell proliferation and differentiation, villus shortening, compromised barrier integrity, and increased intestinal permeability, ultimately reducing nutrient absorption and contributing to symptoms such as diarrhea and weight loss [40]. Our study demonstrated that treatment with SS significantly mitigated 5-FU-induced cellular apoptosis by suppressing the expression of Bax and cleaved-caspase 3 proteins, while also enhancing the expression of the Bcl-2 protein. These results suggest that SS may alleviate 5-FU-induced mucositis by modulating the apoptosis pathway.

5-FU-induced intestinal mucositis is marked by extensive epithelial damage in the intestinal tract, resulting in inflammation, barrier dysfunction, and impaired epithelial tissue regeneration. The process of EMT promotes the loss of intestinal barrier function by converting intestinal epithelial cells from a polar, cohesive phenotype to a mesenchymal phenotype characterized by enhanced migratory and invasive properties [41]. In 5-FU-induced intestinal mucositis, decreased expression of β-catenin and E-cadherin, both epithelial marker proteins, leads to weakened intercellular connections, intestinal barrier disruption, and increased susceptibility to inflammatory factors and pathogen infiltration [42]. Conversely, the upregulation of N-cadherin, a mesenchymal marker protein, indicates a shift toward a mesenchymal phenotype, further aggravating intestinal mucosal damage. EMT contributes to intestinal inflammation through a reinforcing loop. Pro-inflammatory cytokines like TNF-α and IL-6 initiate EMT, which compromises barrier integrity. This process, in turn, releases inflammatory mediators, exacerbating mucosal inflammation [43]. EMT proteins are involved in both the promotion of damage and the facilitation of repair during 5-FU-induced intestinal mucositis. Regulating key markers such as β-catenin, E-cadherin, and N-cadherin can reduce inflammation and enhance mucosal function, providing a critical focus for treatment strategies [44]. This study demonstrated that SS treatment markedly reduced 5-FU-induced EMT, evidenced by decreased N-cadherin expression and increased levels of β-catenin and E-cadherin proteins. These observations highlight the potential of SS in alleviating 5-FU-induced mucositis by targeting the EMT pathway.

Tight junction breakdown in 5-FU-induced intestinal mucositis has been thoroughly examined in preclinical mouse studies. These junctions are vital for maintaining the intestinal epithelial barrier and are formed by proteins like occludin, claudins, and ZO-1. By regulating paracellular permeability, these proteins help preserve intestinal barrier integrity and prevent harmful substances like pathogens and toxins from crossing [45]. After 5-FU treatment, a significant reduction in ZO-1, a key scaffold protein that connects tight junctions with the actin cytoskeleton, was observed, leading to a compromise in the structural integrity of tight junctions and enhanced intestinal permeability. The expression of occludin, a transmembrane protein essential for tight junction function, was significantly reduced following 5-FU exposure, which impairs the barrier integrity of the intestinal epithelium [46]. Additionally, the level of Claudin-1, another crucial tight junction protein, was decreased, leading to heightened intestinal permeability and allowing the influx of inflammatory mediators that aggravate mucosal damage. The reduction in these tight junction proteins is indicative of the toxic effects of 5-FU, where oxidative stress and an increase in ROS disturb the expression of tight junction proteins. Pro-inflammatory cytokines like TNF-α and IL-6 are also implicated in the breakdown of tight junctions [47]. This study reveals that SS treatment significantly attenuated 5-FU-induced tight junction damage, as demonstrated by reduced expression of occludin, claudins, and ZO-1 proteins. These results emphasize the therapeutic potential of SS in reducing 5-FU-induced mucositis via tight junction regulation.

SS has been demonstrated to possess a rich diversity of polyphenolic compounds. The characteristic hydroxyl (OH) groups present in these polyphenolic structures function as effective hydrogen donors, thereby mediating their well-documented antioxidant, anti-inflammatory, and anti-cancer properties [14]. Our previous HPLC analyses have quantitatively verified that SS mycelium extracts contain substantial concentrations of these bioactive polyphenols [14,48,49]. Through comprehensive phytochemical profiling, we identified and quantified eight major phenolic acid constituents [Protocatechuic acid (PCA), protocatechualdehyde, caffeic acid, syringic acid (SA), 2, 5-dihydroxyterephthalic acid (DTA), 3,4-dihydroxybenzalacetone (DBL), inoscavin A, and inoscavin C]. Chromatographic quantification revealed DBL, protocatechuic acid, and inoscavin C as the predominant phenolic components in SS mycelium. These findings provide the chemical foundation for understanding the established biological activities associated with SS extracts, with the relative abundance of these key phenolic compounds suggesting their potential role as primary bioactive constituents.

Emerging research underscores the significant therapeutic advantages of combining polyphenolic compounds with conventional chemotherapeutic agents. PCA demonstrates synergistic effects with 5-FU, achieving dual benefits of dose reduction in 5-FU chemotherapy while simultaneously enhancing apoptotic activity through modulation of p53 and Bcl-2 signaling pathways [50]. Protocatechualdehyde exhibits remarkable cytoprotective properties in human umbilical vein endothelial cells (HUVECs), effectively mitigating oxygen-glucose deprivation/reoxygenation (OGD/R)-induced cellular damage through SIRT1-mediated activation of autophagy and suppression of apoptotic pathways. These findings suggest its promising therapeutic potential for ischemic conditions [51]. Protocatechualdehyde attenuated d-galactose-induced cellular senescence in H9C2 cardiomyocytes by suppressing inflammatory responses, suggesting a potential mechanism through which it mitigates cardiomyocyte aging [52]. Caffeic acid has been demonstrated to exhibit multifaceted pharmacological properties, including antioxidant, antimicrobial, antidepressant, anti-cancer, and anti-inflammatory activities. Specifically, caffeic acid mitigates Fenton reaction-mediated oxidative damage by scavenging free hydroxyl radicals and suppressing their generation [53]. Furthermore, combined treatment with caffeic acid and paclitaxel synergistically inhibits proliferation and induces apoptosis in NSCLC H1299 cells, potentially through modulation of the Bcl-2/Bax signaling axis [54]. SA demonstrates protective efficacy against cisplatin-induced ovarian damage through a multi-mechanistic action by modulation of endoplasmic reticulum (ER) stress responses, suppression of inflammatory pathways, and activation of the Nrf2-mediated antioxidant defense system [55]. 3,4-Dihydroxybenzalacetone (DBL), a distinctive catechol-containing phenylbutanoid, exhibits dual functionality as both a potent reactive oxygen species (ROS) scavenger in oxidative stress models and an inhibitor of amyloid-β aggregation [56]. Furthermore, DBL activates the cytoprotective Nrf2/glutathione axis through PI3K/Akt-dependent signaling, demonstrating anti-inflammatory efficacy in murine models of acute lung injury [57]. Notably, inoscavin A-a characteristic pyrone derivative abundant in Sanghuangporus species-manifests a broad spectrum of bioactive properties, including significant antioxidant capacity, antitumor activity, and potent inhibition of lipoxygenase (LOX) enzymes [58]. Based on these findings, the bioactive polyphenolic components of SS may alleviate 5-FU-induced intestinal damage through multiple mechanisms, including antioxidant, anti-inflammatory, anti-apoptotic, and barrier repair effects. These complementary mechanisms collectively address key clinical challenges associated with 5-FU therapy, such as drug resistance, normal tissue toxicity, and inflammatory complications, while potentially enabling dose reduction. Moreover, the identified structure-activity relationships among these polyphenols—particularly the crucial roles of the catechol group and the α, β-unsaturated carbonyl system—provide a molecular framework for optimizing 5-FU adjuvant therapy. However, further experimental validation is required to elucidate the precise molecular pathways involved.

## 4. Materials and Methods

### 4.1. Preparation of Samples

For this study, *S. sanghuang* mycelium was sourced from Grape King Bio Ltd., located in Taoyuan, Taiwan. The dried SS powders were soaked in 70% ethanol for five days, filtered, and the solvent was evaporated under reduced pressure. The process was repeated four times, and each resulting extract was stored at −80 °C. A prior HPLC analysis identified the phenolic compounds in SS.

### 4.2. Reagents

The study utilized 5-FU, mesalazine, and other reagents, which were supplied by Sigma-Aldrich (St. Louis, MO, USA). The antibodies used, including iNOS (1:1000), COX-2 (1:1000), TLR4 (1:1000), IKK (1:1000), p-IKK (1:1000), IκBα (1:1000), p-IκBα (1:1000), NF-κB (1:1000), p-NF-κB (1:1000), p-ERK (1:1000), ERK (1:1000), p-JNK (1:1000), JNK (1:1000), p-P38 (1:1000), P38 (1:1000), Bax (1:1000), Bcl-2 (1:1000), and caspase-3 (1:1000) were from Cell Signaling Technology (Beverly, MA, USA). Antibodies against catalase (1:1000), SOD1 (1:1500), GPx3 (1:1500), β-catenin (1:1000), E-cadherin (1:1500), N-cadherin (1:1000), Occludin (1:1500), Claudin-1 (1:1500), ZO-1 (1:1000), heme oxygenase-1 (HO-1) (1:2000), Nrf2 (1:1500), and β-actin (1:10,000) were supplied by Abcam (Cambridge, UK, USA). The loading control utilized in this experiment is β-actin.

### 4.3. Animals

This study utilized eight-week-old male BALB/c mice (25 ± 3 g) sourced from BioLASCO Taiwan Co., Ltd. (Taipei, Taiwan). The mice were acclimated to a controlled environment for 3–5 days, with conditions including a 12 h light/dark cycle, 23 °C temperature, and 50% relative humidity. Ethical approval for the study was obtained from the Animal Protection Committee of China Medical University (CMUIACUC-2022-443).

### 4.4. Research Design

A total of 25 male BALB/c mice were used, distributed into five groups (*n* = 5 per group): (1) control group receiving saline (i.p.); (2) group treated with 5-FU (50 mg/kg, i.p.); (3) mesalazine group (10 mg/kg, i.p.) combined with 5-FU (50 mg/kg, i.p.); (4) SS (250 mg/kg oral suspension) in combination with 5-FU (50 mg/kg, i.p.); and (5) SS (500 mg/kg oral suspension) combined with 5-FU (50 mg/kg, i.p.).

From the first day, mice in the control and 5-FU groups received physiological saline by gavage for 10 days, while other groups received their respective treatments. All groups except the control received intraperitoneal 5-FU injections (50 mg/kg) from days 4 to 7. On the 10th day of the experimental timeline, mice were fasted for 16 h before being anesthetized with pentobarbital for blood collection. Serum samples were preserved at −20 °C for biomarker assays, and small intestinal tissues were promptly gathered for pathological staining and Western blot studies (iNOS, COX-2, TLR4, IKK, p-IKK, IκBα, p-IκBα, NF-κB, p-NF-κB, p-ERK, ERK, p-JNK, JNK, p-P38, P38, Bax, Bcl-2, caspase-3, catalase, SOD, GPx3, β-catenin, E-cadherin, N-cadherin, Occludin, Claudin-1, ZO-1, HO-1, Nrf2). Daily assessments of body weight and diarrhea were carried out. Histopathological examinations were performed for all specimens (Figure 1A) [16].

### 4.5. Daily Weight and Diarrhea Monitoring

The body weight and diarrhea severity of each mouse were monitored daily. Two independent investigators confirmed diarrhea scores, and the average score was documented. The grading system was as follows: 0 (normal stool), 1 (soft stool), 2 (slightly wet stool), 3 (wet, unformed stool with moderate perianal staining), and 4 (watery stool with severe perianal staining). Diarrhea severity was evaluated using incidence rates and average scores [16].

### 4.6. Histological Examination 

For histological analysis, 5 µm sections of intestinal tissue were stained with H&E and observed under a Nikon Eclipse TS100 microscope. The severity of intestinal injury was categorized into the following grades: 0 (normal), 1 (mild, less than 25%), 2 (moderate, 25–50%), 3 (severe, 50–75%), and 4 (extensive damage, greater than 75%) [16]. Immunohistochemical (IHC) staining for Ki-67 was carried out to analyze epithelial proliferation in the ulcer healing phase. Slides were deparaffinized and incubated with the Ki-67 antibody at 4 °C for 24 h. Contrast staining was completed with 3,3′-Diaminobenzidine (DAB) (Sigma-Aldrich, Saint Louis, MO, USA). 

### 4.7. Cytokine Assay

Following the instructions provided by the manufacturer, an ELISA kit was used to measure serum concentrations of TNF-α, IL-1β, and IL-6 (BioLegend, San Diego, CA, USA).

### 4.8. Nitrite Assay

The nitrite concentration was determined using the Griess reaction colorimetric method [17]. Briefly, 100 μL of Griess reagent was added to the culture supernatant, mixed, and incubated for 10 min before measuring absorbance at 540 nm using a microplate reader (Molecular Devices, Orleans Drive, Sunnyvale, CA, USA). 

### 4.9. The TBARS (Thiobarbituric Acid Reactive Substance) Assay

Malondialdehyde (MDA), a marker of TBARS and lipid peroxidation, was quantified in small intestinal tissues. The tissues were lysed in an ice-cold buffer and incubated with TBA solution at 90 °C for 45 min, and the resulting MDA-TBA complexes were measured at 532 nm using a spectrophotometer [17]. 

### 4.10. Glutathione (GSH) Assay

DTNB (5, 5′-dithiobis (2-nitrobenzoic acid)) acted as the reagent in the GSH assay. The reaction mix included 100 μL of supernatant, 200 μL of 0.3 M phosphate buffer (pH 8.4), 400 μL of double-distilled water, and 500 μL of Ellman’s reagent [17]. Absorbance at 412 nm was recorded using a spectrophotometer. Protein levels were measured via the Bradford assay, utilizing its dye-binding method (Bio-Rad Laboratories, Hemel Hempstead, UK).

### 4.11. Western Blot Analysis

Small intestinal tissue lysates were prepared by homogenizing in RIPA buffer supplemented with protease inhibitors. Electrophoresis was conducted with primary antibodies, and detection was achieved using HRP-conjugated secondary antibodies (anti-rabbit or anti-mouse IgG). Protein bands labeled with HRP were visualized via chemiluminescent reagents, and imaging was performed with Kodak Molecular Imaging Software (Eastman Kodak Company, Rochester, NY, USA).

### 4.12. Statistical Analysis

The results are represented as mean ± standard deviation (S.D.). To compare the means of the two groups, Student’s *t*-test was performed, and a one-way ANOVA with Scheffé’s post hoc analysis was conducted for multiple comparisons. A *p*-value of less than 0.05 was considered statistically significant.

## 5. Conclusions

This research illustrates how SS modulates oxidative stress in 5-FU-induced mucositis by attenuating histopathological changes in the small intestine. The treatment exhibited strong antioxidant and anti-inflammatory effects, reducing inflammation, apoptosis, EMT, and tight junction disruption. While SS appears to protect against mucositis in the 5-FU-induced oxidative stress model, further investigation is necessary to confirm its broader protective efficacy. In conclusion, SS treatment mitigates oxidative stress and prevents mucositis-associated damage.

## Data Availability

Data are provided within the article.
